# Identification of* bap*A in Strains of* Salmonella enterica *subsp.* enterica* Isolated from Wild Animals Kept in Captivity in Sinaloa, Mexico

**DOI:** 10.1155/2016/3478746

**Published:** 2016-06-09

**Authors:** Gabriela Silva-Hidalgo, Martin López-Valenzuela, Nora Cárcamo-Aréchiga, Silvia Cota-Guajardo, Mayra López-Salazar, Edith Montiel-Vázquez

**Affiliations:** ^1^Pathology Laboratory, Faculty of Veterinary Medicine and Animal Husbandry, Autonomous University of Sinaloa, Boulevard San Ángel s/n, Fraccionamiento San Benito, 80246 Culiacán, SIN, Mexico; ^2^Enteric Bacteriology Laboratory, Institute of Epidemiological Diagnosis and Reference, Francisco de P. Miranda 177, Lomas de Plateros, Álvaro Obregón, 01480 Mexico City, DF, Mexico

## Abstract

*bap*A, previously named* stm2689*, encodes the BapA protein, which, along with cellulose and fimbriae, constitutes biofilms. Biofilms are communities of microorganisms that grow in a matrix of exopolysaccharides and may adhere to living tissues or inert surfaces. Biofilm formation is associated with the ability to persist in different environments, which contributes to the pathogenicity of several species. We analyzed the presence of* bap*A in 83 strains belonging to 17 serovars of* Salmonella enterica *subsp.* enterica* from wildlife in captivity at Culiacan's Zoo and Mazatlán's Aquarium. Each isolate amplified a product of 667 bp, which corresponds to the expected size of the* bap*A initiator, with no observed variation between different serovars analyzed.* bap*A gene was found to be highly conserved in* Salmonella* and can be targeted for the genus-specific detection of this organism from different sources. Since* bap*A expression improves bacterial proliferation outside of the host and facilitates resistance to disinfectants and desiccation, the survival of* Salmonella* in natural habitats may be favored. Thus, the risk of bacterial contamination from these animals is increased.

## 1. Introduction

Biofilms, composed of cellulose, fimbriae, and biofilm-associated protein A (BapA, encoded by* bap*A), are communities of microorganisms that grow in a matrix of exopolysaccharides and can adhere to inert surfaces or living tissues [1]. Biofilm formation is associated with the ability to persist in different environments [2], which contributes to the pathogenicity of several species [3]. It has been shown that bacteria growing in biofilms are more resistant to antimicrobial agents than those growing in planktonic cultures due to their physical structure and the formation of multilayer biofilms [4]. Whereas acute bacterial infections can be eliminated after a brief antibiotic treatment, infections by biofilm-producing bacteria normally fail to be completely eliminated and lead to recurrent infections, which can only be resolved by replacing the initial antibiotic therapy [3].

*Salmonella* are rod-shaped bacteria commonly found in biofilms [5]. This genus includes flagellated, Gram-negative bacteria without spores that thrive in animals' digestive tracts and environments that facilitate long periods of survival, which makes elimination difficult [6].

Fimbriae, or pili, are important for biofilm formation by* Salmonella* [7]. These protein structures recognize a wide range of molecular targets, allowing the bacteria to interact with various surfaces and adhere to specific tissues in the host [8]. For example, type 1 fimbriae are thin, rigid, adhesive structures that express FimH adhesins, which promote bacterial adhesion to and invasion of epithelial cells [9]. Type 1 fimbriae also mediate interactions with abiotic surfaces [9].

*Salmonella* biofilm matrices are composed of cellulose, fimbriae, and BapA. The exopolysaccharide cellulose is a major component of these matrices and plays an important role in the resistance to desiccation, disinfectants, and UV light. Cellulose production is regulated by the union of the cyclic nucleotide c-di-GMP, whose synthesis depends on a family of GGDEF domain-containing proteins [10].

After a* Salmonella* infection, continued elimination of the bacteria in stool gives rise to a chronic asymptomatic carrier. Once excreted into the environment,* Salmonella* can resist dehydration for long periods of time in both stool and food for human or animal consumption [11, 12]. Due to its ability to adhere to many surfaces and resist the action of common disinfectants, the presence of this bacterium in the environment is a public health concern because salmonellosis is a zoonotic disease [13]. Additionally, bacteria in biofilms have greater resistance to antibiotics due to several factors. For example, these bacteria present replicative and metabolic heterogeneity, which affects the action of the antibiotic and the structure of the biofilm, thereby impeding the action of the antimicrobial agent [10].

Understanding the capacity of biofilm formation in this bacterial genus will allow us to establish preventive measures to prevent outbreaks of disease in both animals and humans, particularly those in close contact with infected animals. Identifying the genes involved in bacterial resistance will determine the type of antibiotic therapy necessary to treat animal health problems. Thus, the objective of this study was to detect the presence of* bap*A in* Salmonella* strains isolated from wild animals in captivity.

## 2. Material and Methods

### 2.1. Strains

Eighty-three strains of* Salmonella* spp. belonging to 17 different serovars (Table 1) obtained from enclosures, food, and feces from zoo and aquarium animals in captivity in Culiacan and Mazatlán, Sinaloa, Mexico, were used in the study. All isolates were confirmed through biochemical and serological methods by the Enteric Bacteriology Laboratory, Institute of Epidemiological Diagnosis and Reference (InDRE), DF, Mexico, and maintained on nutrient freezing medium until being tested.* Salmonella* Typhimurium 14028S from the American Type Culture Collection (ATCC) was used as a reference control strain.

### 2.2. Recovery and Purity Verification of Strains

*Salmonella* strains were recovered from preservation medium containing soy broth-glycerol (freezing medium), transferred to trypticase soy broth, and incubated at 37°C for 18 h. The bacterial suspensions obtained were plated on MacConkey and XLT4 agar to confirm the negative reaction of* Salmonella* strains to lactose and to visually analyze the purity of the strains grown at 37°C for 24 h. Inclined tubes containing blood agar base (BAB) were inoculated with confirmed strains until further use.

### 2.3. Bacterial DNA Extraction

DNA was extracted from isolated bacterial strains with a commercial matrix (InstaGene Matrix, Bio-Rad®).

### 2.4. PCR Identification of* bap*A

The oligonucleotide primers for PCR were synthesized according to the published DNA sequences of the* bap*A gene [10] and have, respectively, the following nucleotide sequence: forward, 5′-GCCATGGTGCTGGAAGGCCTGGCGGTT-3′; reverse, 5′-GGTCGACGGGAAGGGTAAAATGACCTTC-3′. Amplification was carried out in a thermocycler (Bio-Rad, MJ Mini Personal Thermal Cycler) with a reaction mixture of 25 *μ*L, which contained 5 *μ*L of template DNA, 1 *μ*L (10 pmol L^−1^) of each of the forward and reverse primers, 12.5 *μ*L PCR SuperMix (22 mM Tris-HCl, 55 mM KCl, 1.65 mM MgCl_2_, 220 *μ*M dGTP, 220 *μ*M dATP, 220 *μ*M dTTP, 220 *μ*M dCTP, and 22 U/mL recombinant Taq DNA Polymerase), and 1.5 *μ*L MgCl_2_ (50 mM). The final volume was prepared with nuclease-free water. The PCR program included an initial denaturation step at 94°C for 5 min followed by 30 cycles of denaturation (94°C for 1 min), annealing (50°C for 45 s), and extension (72°C for 1 min). Final extension was carried out at 72°C for 5 min. Amplification products were separated by submarine gel electrophoresis on 1.5% agarose gel with prestained GelRed (solution at 1 : 10,000) in 0.5x Tris-EDTA electrophoresis buffer. A 100 bp DNA ladder (Bio-Rad) was used as a molecular weight marker. The gels were visualized in Gel Documentation System*™* EZ GelDoc and photographed for analysis.

### 2.5. Statistical Analysis

The frequency of the presence of* bap*A was determined according to the previously reported formula [14]. To determine whether there were significant statistical differences among the different serovars examined, chi-square tests were performed using the epidemiological data analysis program, Epidat 3.1.

## 3. Results and Discussion

**Figure 1:**
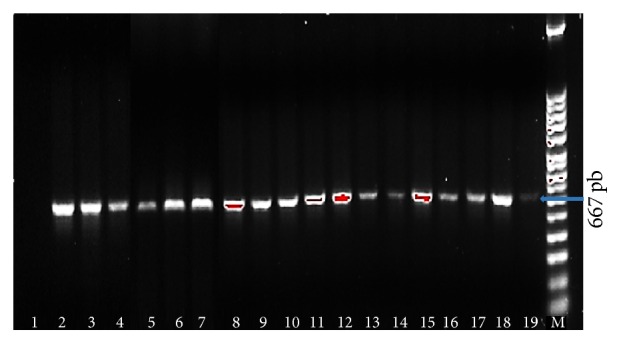
PCR results for the detection of* bap*A from different* Salmonella* serovars. Lane 1: nontemplate control; Lane 2:* Salmonella* Albany; Lane 3:* Salmonella* 3, 10, H: r:-; Lane 4:* Salmonella* San Diego; Lane 5:* Salmonella* Braenderup; Lane 6:* Salmonella* Weltevreden; Lane 7:* Salmonella* Derby; Lane 8:* Salmonella* Oranienburg; Lane 9:* Salmonella* 6, 7, H: en x:-; Lane 10:* Salmonella* Poona; Lane 11:* Salmonella* Saint Paul; Lane 12:* Salmonella* Panama; Lane 13:* Salmonella* Pomona; Lane 14:* Salmonella* Newport; Lane 15:* Salmonella* Enteritidis; Lane 16:* Salmonella* Javiana; Lane 17:* Salmonella* Give; Lane 18:* Salmonella* Agona; Lane 19:* Salmonella* Typhimurium reference strain (ATCC 14028S); Lane M: 100 bp ladder. Thick arrow identifies the 667 bp band of interest.

PCR reactions of the 83 isolates belonging to 17 different serovars with oligonucleotides to* bap*A amplified a product of 667 bp, which corresponds to the expected size of the* bap*A initiator (Figure 1). Importantly, there were no differences detected in this initiator element between different serovars tested. Of the strains analyzed, 65 were isolated from animal feces (mammals, birds, and reptiles), 6 were isolated from* American cockroach* and* Musca domestica*, 2 were isolated from food, 1 was isolated from a biological filter, and 4 and 5 were isolated from enclosures of water and soil, respectively.

All serovars amplified* bap*A, consistent with previous results [10], which suggests that* bap*A is a very conserved gene both between and within different serovars with a high degree of identity (99%) [15]. This conservation offers diagnostic advantages because the presence of* bap*A can be used to identify the* Salmonella* genus in different environments [16].

BapA belongs to a family of large surface proteins involved in bacterial adhesion to various surfaces and maturation of biofilms [17]. The protein, which was previously named Stm2689, plays an important role in the mouse model of intestinal colonization as well as bacterial spread to other organs [18]. In this study, strains were isolated from the stool of wild animals lacking gastroenteric disorders, which suggests that intestinal colonization in these animals is associated, in part, with the presence of the* bap*A gene. Supporting this notion, previous research compared the propensities of* Salmonella* strains with or without* bap*A to colonize the intestine and demonstrated that mutated strains exhibited lower colonization rates than those with wild-type* bap*A [19].

The mechanisms that allow these pathogens to persist in animals' digestive tracts are poorly understood. However, the intestinal persistence of* Salmonella* spp. observed in clinically healthy animals increases the risk of bacterial contamination because* bap*A expression ensures that more bacteria survive outside of the host and retain their infective ability. This allows the bacteria to resist desiccation and the action of the disinfectants; thus, the survival of* Salmonella* in natural habitats may be favored [20, 21]. Further understanding of the mechanisms involved in bacterial intestinal persistence will facilitate the development of innovative strategies that safeguard the public population against salmonellosis, a natural zoonotic disease.

## 4. Conclusions

*bap*A was identified in all 83 strains belonging to 17 different serovars isolated from wildlife in captivity which suggests that it is a highly conserved gene in* Salmonella* and can be targeted for the genus-specific detection of this organism from different sources and diagnostic potentials, which need to be explored. Additionally, most animals that tested positive were asymptomatic carriers. This poses a challenge for professionals in the health care area to overcome, because the capacity of* Salmonella* to survive in many environments suggests that its dissemination will likely continue to increase in the future.

## Figures and Tables

**Table 1:** List of *Salmonella* serovars used in the study.

Identification number	Serovar	Source (# of isolates)
1	Typhimurium	Reference strain

2	Albany	*Leopardus pardalis* (f)^a^, *Panthera leo* (f), *Felis concolor* (f), *Panthera tigris sumatrae* (f), *Panthera tigris tigris *(f), *Lynx rufus* (f), *Ursus americanus* (f), *Hippopotamus amphibius *(f), *Ara macao* (f), *Carassius auratus* (w)^b^, aquatic birds (f), aquatic bird (s)^c^, *Rattus* spp. (f), *Periplaneta americana* (i)^d^, *Musca domestica* (i), raw chicken (F)^e^

3	3, 10, H: r:-	*Hippopotamus amphibius* (f), *Bassariscus astutus* (f), aquatic birds (f), aquatic birds (w), *Cebus apella* (f)

4	San Diego	Aquatic birds (f), aquatic birds (s), *Python regius* (h)^f^, *Rattus *spp. (f)

5	Braenderup	*Mephitis macroura* (f), *Felis concolor* (f), *Panthera tigris* (f), *Procyon lotor* (f), *Ateles geoffroyi* (f)

6	Weltevreden	*Columba flavirostris* (f), *Columba fasciata* (f), *Sus scrofa domestica *(f), aquatic birds (f), aquatic birds (s)

7	Derby	*Cebus apella* (f), *Panthera onca* (f), *Panthera tigris* (f), *Rattus* spp. (f)

8	Oranienburg	*Urocyon cinereoargenteus* (f), *Saimiri sciureus* (f)

9	6, 7, H: en x:-	*Hippopotamus amphibius* (w), *Crocodylus acutus* (w)

10	Poona	Psittaciformes birds (f), *Rattus* spp. (f)

11	Saint Paul	Aquatic birds (f)

12	Panama	*Crocodylus acutus* (w), *Rana* spp. (f)

13	Pomona	*Ramphastos sulfuratus* (f), biological filter

14	Newport	Aquatic birds (f)

15	Enteritidis	Psittaciformes birds (f)

16	Javiana	*Rana* spp. (f)

17	Give	*Iguana iguana* (f)

18	Agona	*Ara* spp. (f)

^a^Feces. ^b^Water. ^c^Soil. ^d^Insect. ^e^Food. ^f^Rectal *Hyssopus*.
